# Ensemble deep model for continuous estimation of Unified Parkinson’s Disease Rating Scale III

**DOI:** 10.1186/s12938-021-00872-w

**Published:** 2021-03-31

**Authors:** Murtadha D. Hssayeni, Joohi Jimenez-Shahed, Michelle A. Burack, Behnaz Ghoraani

**Affiliations:** 1 Department of Computer and Electrical Engineering and Computer Science, Florida Atlantic University, Boca Raton, FL 33431 USA; 2 Icahn School of Medicine at Mount Sinai, New York, NY USA; 3 Department of Neurology, University of Rochester Medical Center, Rochester, NY USA

**Keywords:** Ensemble, Deep models, Parkinson’s disease, Home monitoring, UPDRS, Wearable sensors, Inertial sensors

## Abstract

**Background:**

Unified Parkinson Disease Rating Scale-part III (UPDRS III) is part of the standard clinical examination performed to track the severity of Parkinson’s disease (PD) motor complications. Wearable technologies could be used to reduce the need for on-site clinical examinations of people with Parkinson’s disease (PwP) and provide a reliable and continuous estimation of the severity of PD at home. The reported estimation can be used to successfully adjust the dose and interval of PD medications.

**Methods:**

We developed a novel algorithm for unobtrusive and continuous UPDRS-III estimation at home using two wearable inertial sensors mounted on the wrist and ankle. We used the ensemble of three deep-learning models to detect UPDRS-III-related patterns from a combination of hand-crafted features, raw temporal signals, and their time–frequency representation. Specifically, we used a dual-channel, Long Short-Term Memory (LSTM) for hand-crafted features, 1D Convolutional Neural Network (CNN)-LSTM for raw signals, and 2D CNN-LSTM for time–frequency data. We utilized transfer learning from activity recognition data and proposed a two-stage training for the CNN-LSTM networks to cope with the limited amount of data.

**Results:**

The algorithm was evaluated on gyroscope data from 24 PwP as they performed different daily living activities. The estimated UPDRS-III scores had a correlation of $0.79\, (\textit{p}<0.0001)$ and a mean absolute error of 5.95 with the clinical examination scores without requiring the patients to perform any specific tasks.

**Conclusion:**

Our analysis demonstrates the potential of our algorithm for estimating PD severity scores unobtrusively at home. Such an algorithm could provide the required motor-complication measurements without unnecessary clinical visits and help the treating physician provide effective management of the disease.

**Supplementary information:**

The online version contains supplementary material available at 10.1186/s12938-021-00872-w.

## Background

Parkinson’s disease (PD) is a key chronic, progressive neurological disorder. It often occurs in older people and impacts motor as well as non-motor activities of the patients [1]. People with PD (PwP) at mid- and advanced stages of the disease experience motor complications such as troubling motor fluctuations [2]. Motor fluctuations are experienced as levodopa, the main PD medication, wears off between doses, and the PD symptoms reappear [3]. At this stage of the disease, an iterative therapeutic adjustment is needed to manage the motor fluctuations through multiple clinical visits. As part of these visits, part III of the Unified Parkinson Disease Rating Scale (UPDRS III) is assessed by a neurologist to measure the severity of PD motor complications such as tremor and bradykinesia (i.e., slowness of voluntary movements) [4]. UPDRS-III score, besides history-taking and subject reports, is the main contributing factor to a successful therapeutic adjustment. Wearable inertial sensors have the potential to capture complex body movements related to PD symptoms, thus, they can be used to assess UPDRS III. The significance of continuous at-home assessment of UPDRS III is providing a tool for longitudinal monitoring of daily motor fluctuations [5] and managing PD medications [6]. It will limit the need for in-person clinical examinations of PwP and reduce exposure to risk of infection from infectious agents such as COVID-19 [7].

To assess UPDRS III, PwP are required to perform several tasks, such as sitting at rest, finger and toe-tapping, hand movement, gait, and arising from a chair. A home-based system for continuous and unobtrusive PD severity assessment using wearable sensors has to score UPDRS III without requiring the patients’ active engagement. However, we cannot achieve such a system without addressing two main limitations in the existing work. First, work in this area has been mostly focused on estimating the severity of each of the PD symptoms separately, instead of the total UPDRS-III score. For example, Griffiths et al. [8] and Sama et al. [9] estimate bradykinesia severity and then use the estimated value as the UPDRS-III score. Similarly, Pan et al. [10] and Dia et al. [11] estimate tremor severity instead of UPDRS III directly. Pulliam and colleagues estimate tremor [12] and bradykinesia subscore [13]. Second, existing methods to estimate the UPDRS-III score are obtrusive as they require subjects’ active engagement to perform some specific tasks to elicit PD symptoms. For example, Giubert et al. [14] require sit-to-stand task to estimate UPDRS III. Rodriguez et al. [15] and Zhao et al. [16] propose an algorithm to estimated UPDRS III based on gait. Parisi et al. [17] require the patients to perform the UPDRS-III tasks of gait, leg agility, and sit to stand. In another work [18], an approach is developed to estimate mobile PD score (mPDS) that measures PD severity using a smartphone application as subjects perform five specific tasks (gait, balance, finger tapping, reaction time, and voice). However, the work of Pissadaki et al. [19] shows that complex body movements during ADL mostly can be decomposed into movement primitives performed during the UPDRS-III clinical exams. We, therefore, hypothesize that effective machine-learning algorithms can estimate the UPDRS-III total score unobtrusively during ADL without the limitations of the current approaches.

Most of the methods in the papers mentioned above are based on hand-crafted features and traditional machine learning. However, recent work based on deep learning has shown to outperform the traditional methods in assessing different aspects of PD disease. For example, Hammerla and colleagues show that a sequence of Restricted Boltzmann Machines provides a better generalization than the traditional machine-learning methods used for PD medication state detection [20]. Zhao et al. compare the performance of Short-Term Memory (LSTM), Convolutional Neural Network (CNN), and dual-channel deep model with traditional methods and show a high performance using the LSTM networks for PD severity estimation [16]. Artificial Neural Network has been shown to outperform the traditional methods for classifying PD severity [21] or estimating UPDRS III [22]. In a recent work, we also show that LSTM provides promising results for detecting PD motor fluctuations during a variety of daily living activities [23]. Hence, in the present work, we take advantage of deep learning for data-driven feature extraction from raw signals and learning temporal patterns.

Our objective in this paper is to develop a novel algorithm based on deep learning to continuously estimate UPDRS III from the complex ADL movements collected during the subjects’ free body movements. Our algorithm is based on the ensemble of three deep models. One is an LSTM network with hand-crafted features trained using transfer learning with an activity recognition dataset. The other two models are based on data-driven features from raw signals and their time–frequency representations. We also proposed a two-stage training method to address challenges of training deep-learning models with limited data. For comparison purposes, we also implemented a traditional model based on Gradient Tree Boosting in this paper.

## Results

The developed algorithm for estimating UPDRS III is based on free movement gyroscope data collected from the most affected wrist and ankle using wearable sensors. We ensured the deep models were diverse and achieve better performance by training them on hand-crafted features that represent experts’ knowledge about the presentations of PD symptoms on body movements and data-driven features extracted from raw signals and their time–frequency representations. One deep-learning model was a dual-channel LSTM used with hand-crafted features. This proposed structure was based on our preliminary work indicating that a dual-channel LSTM network outperforms a single-channel LSTM for estimating UPDRS-III score [24]. The other two models were used with raw signals: a 1D CNN-LSTM network for raw signals and a 2D CNN-LSTM network for the time–frequency representation of the raw signals. We utilized transfer learning for the hand-crafted LSTM network to cope with the limited amount of data and proposed a novel two-stage training for the data-driven networks.

For our evaluation purposes, we used a dataset of 24 PwP as they performed a variety of ADL in a clinical setting. Fifteen of the subjects completed four rounds of ADL intermittently with a 1-h gap for about 4 h, and the other nine subjects performed ADL continuously for about 2 h. UPDRS III was performed before each round for the 15 subjects and at the beginning and end of the other subjects’ experiments. First, we evaluated the performance of each deep-learning model for estimating UPDRS III separately. We also compared their performance against traditional machine learning based on Gradient Tree Boosting. Next, we evaluated the performance of the ensemble of different combinations of deep-learning models.

The proposed models generated a UPDRS-III score for each round of ADL that was about 4 min for 15 subjects and 10 min for the other nine subjects. For the ensemble algorithm, the estimated UPDRS-III scores using the individual models were averaged. All the training and testing steps were performed in subject-based, leave-one-out cross-validation (LOOCV). In each of the 24 cross-validation iterations, the data of one subject were used for testing, and the data of the other subjects were used for training. In addition, an inner split was applied to the training data to select a random 20% for validation. Pearson correlation ($\rho$) and Mean Absolute Error (MAE) were used to evaluate the developed network. A high correlation $\rho$ and low MAE indicate a close estimation of UPDRS III when compared to the gold-standard scores.

Table 1 reports the performance of each of the individual deep models in comparison with Gradient Tree Boosting and the performance of the ensemble of two or three deep models. Among the single models, CNN-LSTM using raw signals had the highest $\rho$ of $0.70 \, (\textit{p}< 0.001)$. Gradient Tree Boosting resulted in the least $\rho$ and MAE performance. Note that transfer learning improved the performance of the model with the hand-crafted features from $\rho$ of 0.62 to 0.67 and MAE of 7.50–6.85. Ensemble of the two deep-learning models improved the single models’ performance by increasing $\rho$ and reducing MAE. The best performance was achieved by the ensemble of the three deep models with $\rho = 0.79\, (\textit{p}< 0.001)$ and MAE = 5.95.

**Table 1:** The LOOCV testing correlation ($\rho$) and MAE of the proposed deep models and Gradient Tree Boosting are reported for single models and the ensemble of two or three models of the deep models

Method		$\rho$	MAE
Single	Gradient Tree Boosting	0.61	7.85
	Dual-channel LSTM, hand-crafted features	0.62	7.50
	Dual-channel LSTM, hand-crafted features, with transfer learning	0.67	**6.85**
	1D CNN-LSTM for raw signals	**0.70**	6.93
	2D CNN-LSTM for time–frequency data	0.67	7.11
Ensemble	Dual-channel LSTM, hand-crafted features, with transfer learning 1D CNN-LSTM for raw signals	0.77	6.04
	Dual-channel LSTM, hand-crafted features, with transfer learning 2D CNN-LSTM for time–frequency data	0.76	5.99
	1D CNN-LSTM for raw signals 2D CNN-LSTM for time–frequency data	0.74	6.54
	Dual-channel LSTM, hand-crafted features, with transfer learning 1D CNN-LSTM for raw signals 2D CNN-LSTM for time–frequency data	**0.79**	**5.95**

The correlation was significant for all models (i.e., $\textit{p}< 0.001$)

**Fig. 1:**
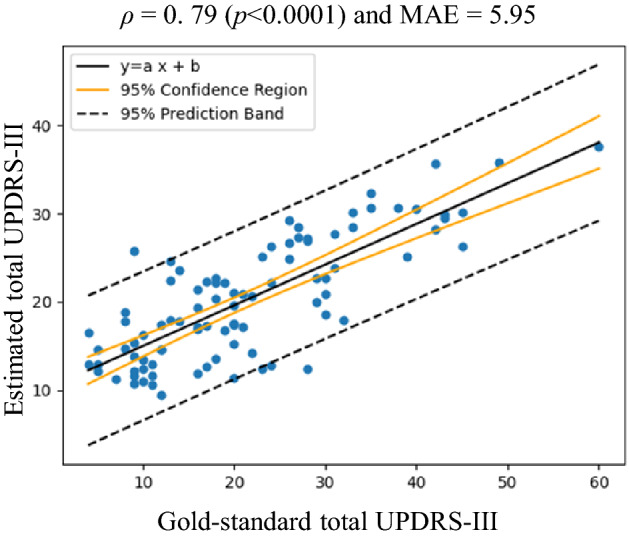
The estimated total UPDRS-III scores using the ensemble of the three deep models vs. the gold-standard total UPDRS-III scores

**Fig. 2:**
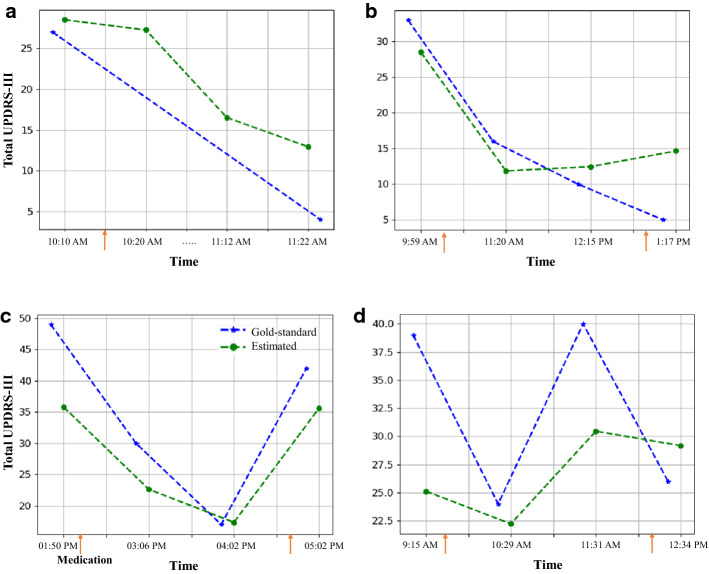
The ensemble model estimations of UPDRS III over time vs. the gold-standard UPDRS III for four PwP. **a** and **b** PwP who experienced an improvement in their PD symptoms. **c** A patient who experienced the return of PD symptoms before taking the next dose of medication. **d** A similar behavior; however, it also shows a reduction in the symptoms after receiving the second dose. Note that the data used for UPDRS-III estimation were from either before or after the UPDRS-III assessment. As a result, the estimated and gold-standard time points do not coincide. Patient A performed only two UPDRS III assessment. The red arrow indicates medication intake

The estimated total UPDRS-III scores using the three deep models’ ensemble vs. the gold-standard total UPDRS-III scores is shown in Fig. 1. Figure 2 shows the ensemble model estimations of UPDRS III over time vs. the gold-standard UPDRS III for four PwP. The examples shown in A and B are from PwP with steady improvement in PD symptoms after medication intake. The two examples in C and D are for PwP who experienced reappearance of their symptoms before their next medication intake (i.e., motor fluctuations). In all the cases, the algorithm follows the change in UPDRS III with a good correlation. Additional file 1: Figure S1 and S2 show the ensemble model estimations of UPDRS III over time vs. the gold-standard UPDRS-III scores for all the 24 PwP.

**Fig. 3:**
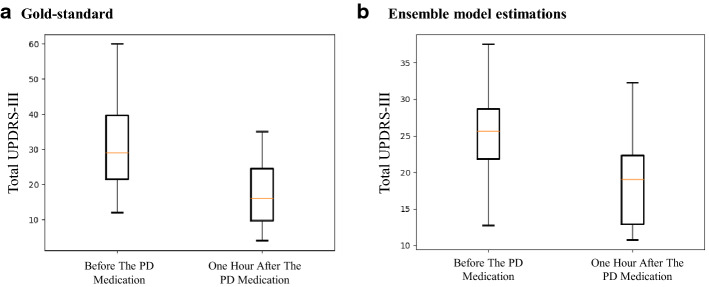
The total UPDRS-III scores before and 1 h after taking the PD medications from gold-standard measurements (**a**) and the ensemble model estimations (**b**). Both the gold-standard and estimated UPDRS-III scores show a significant drop after PD medication intake $(\textit{p} < 0.001)$

As shown in Fig. 3a, a reduction in the gold-standard UPDRS-III score is expected up to 1 h after the medication intake. We investigated whether the estimated scores from the ensemble model show similar behavior in the UPDRS III scores as the medication kicks in. The results are shown in Fig. 3b. Both the gold-standard and estimated UPDRS-III scores indicate a significant difference after patients take their PD medications as confirmed by a paired t-test with $\textit{p} < 0.001$. In addition, Additional file 1: Figure S3 shows the box-plots of the total UPDRS-III scores from the single models before and 1 h after taking the PD medications. The estimated UPDRS-III scores from all models indicate a significant difference after patients take their PD medications as confirmed by a paired t-test with $\textit{p} < 0.01$.

## Discussions

### Unobtrusive estimation of UPDRS III

We hypothesized that advanced machine-learning algorithms could estimate UPDRS III from patients’ free body movements as collected using two wearable sensors placed on the upper and lower extremities. Our analysis indicated such a possibility with a high correlation of $\rho = 0.79\, (\textit{p}< 0.001)$ and low MAE = 5.95 when using an ensemble of three deep-learning models. Most of the existing work for UPDRS III estimation requires PwP’s active engagement to perform the specific tasks used in the UPDRS-III procedure [15, 17, 18, 25]. Unlike these approaches, our algorithm could estimate UPDRS III as the patients performed a variety of ADL without the need for performing constrained tasks. As a result, our system has the potential to be translated into unobtrusive home-based monitoring for continuous assessments of UPDRS III. It can track changes in motor fluctuations due to the medication wearing-off effect, as shown in Fig. 2, and tracking the response to medication, as shown in Fig. 3.

Another interesting observation from our analysis is our algorithm’s ability to estimate UPDRS III scores despite the following challenges. First, the UPDRS-III score is measured by assessing the face/head, neck and all four extremities, but our system is based on only two sensors placed on the wrist and ankle of the most affected side of the body. Second, total UPDRS III includes items representing symptoms measures such as rigidity, speech, and facial expression that cannot be captured by wearable motion sensors. However, our ensemble model captured the dependencies between these items [26, 27] and achieved a high correlation. However, these challenges impacted the estimation MAE, and thus our model was only comparable to the minimal clinically important difference in UPDRS III.

### Comparison to related work

A review of the methods proposed for estimating the severity of PD is shown in Table 2. Comparing our algorithm to task-dependent approaches (i.e., obtrusive methods) [15, 17, 18, 25] indicates that our method provides comparable performance with the advantage of not constraining PwP’s activities. For example, it has a better correlation than Ref. [15] with -0.56, equal or slightly lower than Refs. [17, 25] and lower than Ref. [18] with 0.88. However, it is worth mentioning that the work in Ref. [18] is based on performing a series of tasks using a smartphone application, while ours is solely based on movement data patterns.

Comparing our algorithm to unobtrusive methods [8, 13, 28] shows that our model outperforms Ref. [8] with 0.64 even though they only estimated bradykinesia. Our algorithm performs slightly lower than Refs. [13, 28]. Our careful analysis of the work in Ref. [13] indicates that the results reported by Pulliam et al. [13] were not based on LOOCV. The authors instead developed multiple linear regression models to estimate tremor, bradykinesia, and dyskinesia, and then designed a radar chart reporting the severity and duration of these symptoms. The correlation between the radar chart area and UPDRS III was 0.81 when the models fit all the data. The authors did not report their algorithm’s performance on a held-out set or in a cross-validation fashion; thus, their model’s generalizability is not comparable to ours. Another limitation of Pulliam’s work et al. [13] is the challenge involved with interpreting the range of the estimated area to the clinically meaningful range used for the UPDRS III as their estimated range is different from the clinically meaningful range of the UPDRS III. Other limitations are that they included dyskinesia severity for estimating UPDRS III. However, dyskinesia is a side effect of taking levodopa and not a PD symptom and is not included in the UPDRS-III assessments. Abrami et al. [28] developed an unsupervised algorithm based on clustering and Markov-Chain. They applied a multi-dimensional scaling algorithm to estimate each subject’s daily UPDRS-III score as the sum of tremor, bradykinesia, and gait items for each day. They reported a high $\rho ^2$ of 0.64 in clinic, but a significantly lower $\rho ^2$ of about 0.43 at home. Our algorithm performed better ($\rho ^2$ = 0.58) than their method at home ($\rho ^2$ = 0.43) but slightly lower in clinic ($\rho ^2$ = 0.64). However, their estimation does not include UPDRS-III items such as rigidity, voice, and facial expressions. Their method also performed better when patients performed more tasks, which was the case in the clinic, where they performed more than nine scripted tasks. At home, people performed fewer tasks in a short time, which could be the reason for the lower performance at home. In addition, there is no information about the ability of their method for hourly estimation of UPDRS III.

**Table 2:** Proposed methods in the literature for estimating the severity of PD represented by UPDRS III

Reference	PwP	Sensors location	Method	Unobtrusive	Estimated metric	Gold-standard label	Validation Method	*r*	MAE
Griffiths et al. [8]	25	Wrist	Statistical approach	Yes	Bradykinesia score	UPDRS III	Held-out testing set	0.64	18
Parisi et al. [17]	34	Chest, left and right thighs	Multiple k-Nearest Neighbors models to estimate LA, S2S and G.	No (task-dependent)	Sum of leg agility, sit-to- stand and gait items of UPDRS III	Sum of leg agility, sit-to- stand and gait items of UPDRS III	LOOCV	0.79	-
Rodriguez-Molinero et al. [15]	75	Waist	Linear regression	No (task-dependent)	Gait item of UPDRS III	UPDRS III	Held-out testing set	-0.56	-
Pulliam et al. [13]	13	Wrist and ankle	Multiple linear regression models to estimate tremor, bradykinesia and dyskinesia	Yes	Radar chart of PD tremor, bradykinesia and dyskinesia	UPDRS III	-	0.81	-
Zhan et al. [18]	152	Smartphone	Rank-based framework for disease severity score [30]	No (task-dependent)	Mobile PD score	UPDRS III	Held-out testing set	0.88	-
Abrami et al. [28]	60	Both wrists	Clustering and Markov-Chain	Yes	Multi-dimensional scale	Sum of tremor, bradykinesia and gait items of UPDRS III	Held-out testing set	$r^2$ = 0.64 in clinic $r^2$ = 0.43 at home	
Butt et al. [25]	64	Wrist, fingers, and foot	Adaptive neuro- fuzzy inference system	No (task-dependent)	UPDRS III	UPDRS III	Tenfold cross validation	0.81	-
The developed approach in this study	24	Wrist and ankle	Ensemble of dual- Channel LSTM, CNN-LSTM using raw signals and CNN-LSTM using spectrogram	Yes	UPDRS III	UPDRS III	LOOCV	0.79	5.95

### The advantage of deep learning

The dual-channel LSTM developed in our preliminary work [24] provides only slightly higher performance than Gradient Tree Boosting with a 0.62 correlation vs. 0.61. However, transfer learning from the activity recognition dataset improves performance by providing a 10% higher correlation and 13% lower MAE when compared to Gradient Tree Boosting. This behavior indicates that temporal dependencies captured by the first two LSTM layers using hand-crafted features extracted from healthy subjects are beneficial to UPDRS-III estimation.

Another observation is that both the 1D and 2D CNN-LSTM networks outperform Gradient Tree with 0.70 and 0.67 correlation, respectively with greater than 10% increase in correlation, and 6.93 and 7.11 MAE, respectively, with a decrease of greater than 9% in MAE. These networks achieve comparable performance to the dual-channel LSTM with hand-crafted features, which means CNN could extract relevant data-driven features.

We also observe that the ensemble of the models based on hand-crafted and data-driven features improves the performance. The ensemble of multiple models is known to improve the regression results if the models solve different aspects of the given problem [29]. Hence, we can conclude that the trained deep models are diverse and learn different views of the motion signals (i.e., hand-crafted features, data-driven features from raw signals and from the time–frequency data), and therefore, are necessary for successful UPDRS-III estimation.

### Limitations and future work

**Fig. 4:**
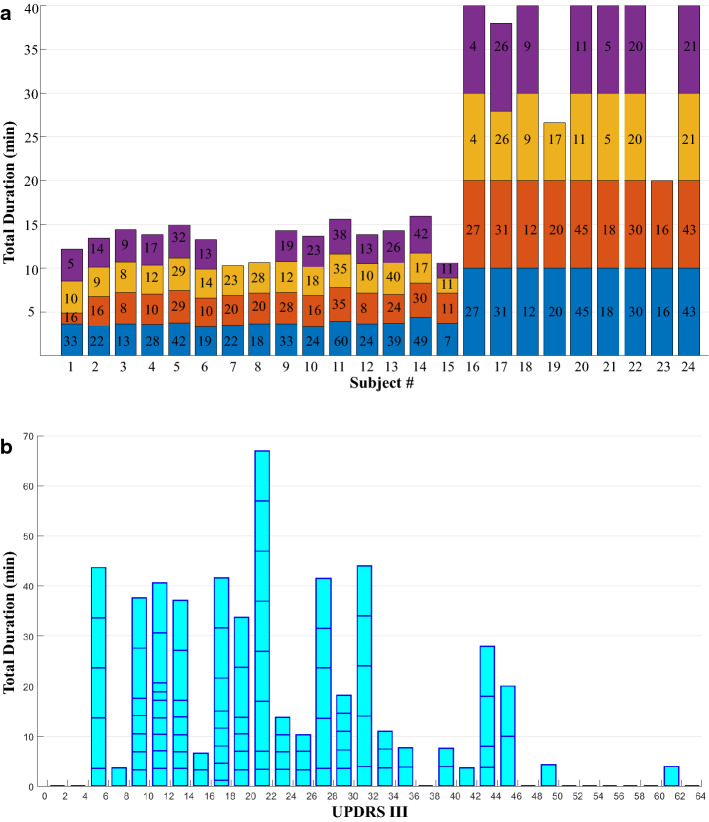
The structure of the processed data from the 24 subjects. **a** The rounds’ duration and their UPDRS-III scores are shown for each subject. The color of each bar represents a round of data, and the height of the bar indicates the duration of the round. Each bar’s number shows the UPDRS-III score as determined by the nearest UPDRS assessment to the round. **b** The rounds’ distribution is displayed based on their UPDRS-III scores

Our algorithm provides overall high performance for UPDRS-III estimation using patients’ free body movement data. However, we notice that the model underestimates high UPDRS-scores, as shown in Fig. 1. This is because of the imbalanced data distribution as there are only nine rounds of ADL with the UPDRS III score of higher than 40, and only one is above 50 (see Fig. 4b). Parisi et al. [17] reported a similar limitation due to the imbalance distribution of their training data toward the mean score of UPDRS III. Collecting more data in a home setting with a uniform data distribution is expected to improve our algorithm’s performance further and consists of the main aspect of our future work.

## Conclusions

We developed a novel algorithm to provide a continuous and unobtrusive estimation of the UPDRS-III score using free-body motion data recorded from two wearable sensors. The novelty aspect our proposed approach is combining both expert knowledge in the field by extracting hand-crafted features with data-driven knowledge using deep learning to extract features from raw temporal and time–frequency signals. To the best of our knowledge, we proposed the first ensemble algorithm based on three deep models to provide a continuous and unobtrusive estimation of the UPDRS-III score using free-body motion data recorded from two wearable sensors. In addition, we utilized transfer learning from an activity recognition dataset for the model using the hand-crafted features and a two-stage training for the models dealing with the raw data. The models were evaluated and compared using the sensor data of 24 PD subjects. Subject-based, LOOCV demonstrated that the three deep models’ ensemble provided a high correlation of $\rho =0.79\, (\textit{p}<0.0001)$ and a low MAE of 5.95, indicating that each model learns different aspects of the PD motor complications from the movement data. We compared our algorithm with the existing work in the literature and discussed the different advantages of our developed algorithm as providing relatively high performance while providing an unobtrusive estimation of UPDRS III from ADL; direct estimation of UPDRS III instead of estimating the symptoms such as tremor or bradykinesia and then delivering it as the estimated of the UPDRS III; estimation of total UPDRS III without removing any of the items such as rigidity or facial expression; and estimation of the clinically known range of UPDRS III instead of providing a new metric, which requires interpretation. Our future work includes evaluation of more training data collected from an at-home setting to further increase the performance of our algorithm.

## Methods

In this section, we first describe the PD dataset [13, 31] that was used for evaluating the developed models. We also provide a brief description of the Physical Activity Monitoring Dataset (PAMAP2) [32] that was used for transfer learning of the deep model with hand-crafted features. Next, we describe signal segmentation and extraction of the hand-crafted features. Finally, we describe the proposed deep models.

### Datasets

#### Collection of PD Data

A protocol was designed to record motion data of 24 PwP with idiopathic PD as they performed a variety of ADL [13, 31]. A summary of patient characteristics is shown in Table 3. The age average was 58.9 years, and the age range was between 42 and 77 years. Fourteen of the PWP were female and ten were male. The average of the disease duration was 9.9 years, and the range was between 4 and 17 years. The UPDRS-III average was 29.7 before taking PD medications and 17.3 1 h after taking PD medications. The institutional review board approved the study, and all patients provided written informed consent.

**Table 3:** Subject demographics. LEDD stands for Levodopa Equivalent Daily Dose. Values are presented as number or mean ± standard deviation

Number of subjects	24	UPDRS III before medication	29.7±12.3
Age (y)	58.9±9.3	UPDRS III after medication	17.3±8.4
Sex (M, F)	14,10	LEDD (mg)	1251±468
Disease duration (y)	9.9±3.7		

Two wearable sensors (Great Lakes NeuroTechnologies Inc., Cleveland, OH) consisting of triaxial gyroscope and accelerometer were mounted on the most affected wrist and ankle to collect the motion data at a sampling rate of 64 Hz. The participants stopped their PD medication the night before the experiment and started the experiments in their *medication OFF* states. Fifteen of the subjects performed various ADL in four rounds spanned for 4 h. The ADL were cutting food, unpacking groceries, grooming, resting, drinking, walking, and dressing. The time of each activity trial ranges between 15 and 60 s, and each round was about 2–4 min. The subjects were asked to perform the ADL at self-pace, and no training was provided. After the first round, the subjects resumed their routine PD medications. 20 trials of activities were missing due to unsuccessful data collection. In addition, two subjects performed three rounds since they started the experiment in their *medication ON* states. The total duration of each round for all the 15 subjects is shown in Fig. 4a.

The other nine subjects cycled through multiple stations (such as laundry room, entertainment station, snack, and desk work) in a home-like setting while engaging in unconstrained activities. Next, the subjects resumed their routine PD medications. Later, when the medicine kicked in (as confirmed by a neurologist), the subjects repeated the same ADL or cycled through the stations in their *medication ON* states. For these nine subjects, the recording was continuous for about 2 h. Later, rounds of 10 min were segmented close to UPDRS-III assessments as shown in Fig. 4a.

Concurrently, the clinical examinations were performed by a neurologist to measure and record the subjects’ UPDRS-III scores. Four rounds of UPDRS-III assessment were performed for 15 subjects at the beginning of every hour of the experiment. Two rounds of UPDRS-III assessment were performed at the beginning and end of the experiment for the other nine participants. In each assessment, 27 signs of PD were scored on a 0–4 scale for different body parts and both sides; thus, the range of UPDRS III was 0–108, the sum of scores from the 27 signs.

#### Physical activity monitoring dataset

PAMAP2 is a public dataset of motion signals recorded using two wearable sensors while nine healthy subjects performed various ADL. The subjects were 27.22 ± 3.31 years old, with eight males and one female. The wearable sensors contained triaxial gyroscopes and accelerometers with a 100 Hz sampling rate and were mounted on the dominant side’s arm and ankle. The recorded ADL included 12 protocol activities such as lying, sitting, standing, walking, watching TV, and working on a computer. We used this dataset for transfer learning of the deep-learning models. The reason for selecting this dataset was the availability of the gyroscope signals and the similarity in the sensor placement locations with our PD dataset.

### Data preprocessing

For both datasets, we used only angular velocity signals generated from the gyroscopes. We found experimentally that the gyroscope sensor performs better than accelerometer sensors in estimating UPDRS III, which is in agreement with the finding of Dia et al. [11]. In addition, using one sensor type decreased the computation power and time required to train and test the models because of the reduction in data dimensionality. The energy consumption of gyroscopes is higher than that of accelerometers, which can constrain the long-term recording [33]. However, the availability of devices with long battery life can avoid this issue. The collected signals were filtered to eliminate low and high-frequency noises using a bandpass FIR filter with a 3 dB cutoff frequency (0.5–15 Hz).

For the PD dataset, we excluded the data recorded during the UPDRS-III examination from our analysis to ensure that the developed model will not benefit from the UPDRS III-specific tasks that elicit PD symptoms. Next, 2–4 rounds of data with a maximum duration of 10 min (i.e., maximum $N_S$ samples) were selected from each subject’s recordings. Fig. 4a demonstrates the number and duration of rounds as well as the corresponding UPDRS-III score for all the subjects. A total of 91 rounds (${N_R}$) were selected to form the set $\mathcal {D}=\{ (X^{(r)},y^{(r)}) \}_r^{N_R}$ $(X^{(r)} \in \mathbb {R}^{N_S^{(r)} \times 6}$, $y^{(r)} \in \mathbb {R})$ where $X^{(r)}$ denotes the motion time-series data in round *r* with $N_S^{(r)}$ as the number of samples in this round, and $y^{(r)}$ denotes the UPDRS-III score for round *r*. The set was used to train and test the developed algorithm using LOOCV. The distribution of these rounds based on the assessed UPDRS III is shown in Fig. 4b. Similarly for PAMAP2 dataset, 1-min rounds of data were selected from each subject’s recordings after down-sampling the signals to 64 Hz. Each round included one activity. A total of 455 rounds were selected to form the set $\mathcal {D}$ for PAMAP2 dataset.

### Segmentation

**Fig. 5:**
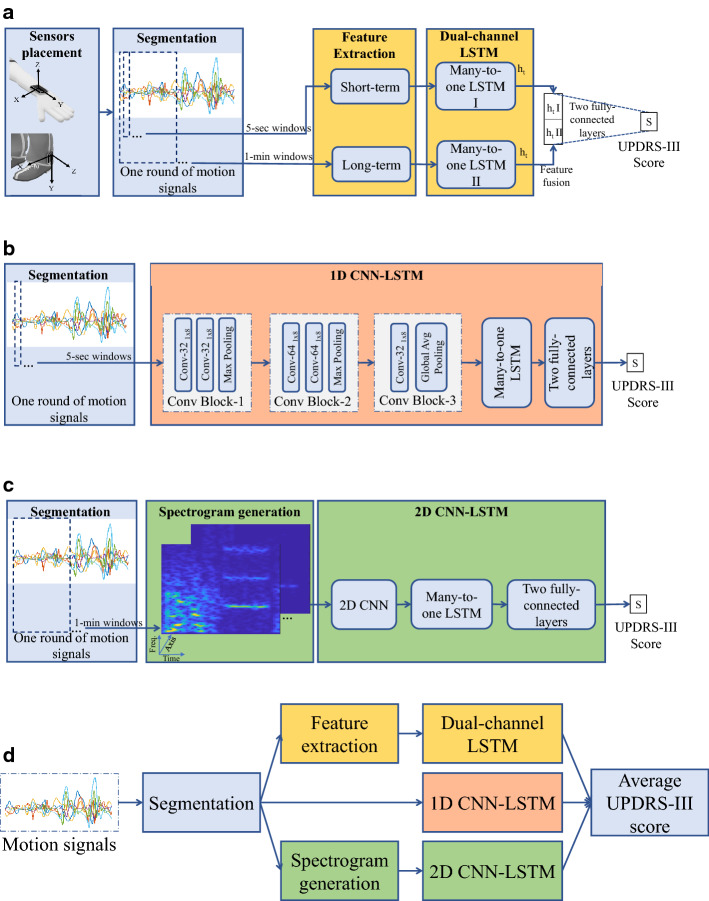
The architectures of the proposed deep models to estimate UPDRS-III score. **a** Dual-channel LSTM network to estimate UPDRS III from hand-crafted features. **b** 1D CNN-LSTM network to estimated UPDRS III from raw signals. Each convolutional layer is followed by a ReLU activation layer. Convolutional Block-2 was repeated to increase the depth of the CNN network. **c**. 2D CNN-LSTM network to estimate UPDRS III from time–frequency representations. The spectrogram of each 1-min window is the input to the CNN network. **d** The overall architecture of the proposed ensemble model

The PD symptoms have both short- and long-term representations on the body movements. Therefore, there is a need for features extracted from both short and long-term duration of the motion signals [34, 35]. Hence, we used 5-s windows to segment the signals for short-term features, and 1-min windows for long-term features. The segmentation process is shown in Fig. 5a.

### Feature extraction

We extracted $N_{SF}=$26 short- and $N_{LF}=$32 long-term features from each segment of the data. First, 39 short-term features were extracted from the three (*x*, *y*, *z*) axes’ signals of the wrist and 39 from the ankle sensor (i.e., segmented *X*). The short-term features were selected to capture high-frequency symptoms such as tremor. They consisted of 4–6 Hz signal power (3 features = x3), percentage power of frequencies > 4 Hz (x3), 0.5–15 Hz signal power (x3), amplitude and lag of the first auto-correlation peak (x6), number and sum of auto-correlation peaks (x6), spectral entropy (x3), dominant and secondary frequencies and their powers (x12), cross-correlation (x3) between *x* and *y*, *x* and *z* and *y* and *z* axes. The details of these features were provided in our previous work [36]. This step provided a total of 78 features from the three axes of the wrist and ankle sensors. Next, the features were averaged across the three axes to get $N_{SF}=$ 26. To conclude, a feature vector ($\vec {fv} \in \mathbb {R}^{N_{SF}}$) was extracted from each 5-s window and provided a set of $\mathcal {D}_{S}=\{ (S^{(r)},y^{(r)}) \}_r^{N_R}$ $(S^{(r)} \in \mathbb {R}^{{N_{Ws}^{(r)}} \times N_{SF}}$, $y^{(r)} \in \mathbb {R})$ where $S^{(r)}=[\vec {fv}_1\vec {fv}_2...\vec {fv}_{N_{Ws}^{(r)}}]$, and $N_{Ws}^{(r)}$ was the number of 5-s windows in round *r*.

Similarly, 48 long-term features were extracted from the three (*x*, *y*, *z*) axes’ signals of the wrist and 48 from the ankle sensor (i.e., segmented *X*). The long-term features were selected to capture low-frequency symptoms such as bradykinesia. These features were average jerk (x3), velocity peak-to-peak (x3), 1–4 Hz signal power (x3), 0.5–15 Hz signal power (x3), Shannon entropy (x3), standard deviation (x3), number and sum of auto-correlation peaks (x6), Gini index (x3), sample entropy (x3), mean (x3), skewness (x3), kurtosis (x3), spectral entropy (x3), dominant frequency and its power [36] (x6). Next, the features were averaged across each axes to get $N_{SF}=$ 32. To conclude, a feature vector ($\vec {fv} \in \mathbb {R}^{N_{LF}}$) was extracted from each 1-min window and provided a set of $\mathcal {D}_{L}=\{ (L^{(r)},y^{(r)}) \}_r^{N_R}$ $(L^{(r)} \in \mathbb {R}^{N_{Wl}^{(r)} \times N_{LF}}$, $y^{(r)} \in \mathbb {R})$, where $L^{(r)}=[\vec {fv}_1\vec {fv}_2...\vec {fv}_{N_{Wl}^{(r)}}]$, and $N_{Wl}^{(r)}$ was the number of 1-min windows in round *r*.

### Regression models for UPDRS-III estimation

In our preliminary work, we explored two different architectures based on a single-channel and dual-channel LSTM of hand-crafted features and showed that the latter provides superior performance [24]. In this section, we first describe an extension to that model by applying transfer learning using PAMAP2 dataset. Next, we develop a new 1D and 2D CNN-LSTM models using raw motion signals and their time–frequency representations, respectively. The proposed ensemble model is described next. Lastly, Gradient Tree Boosting is described as a traditional machine learning method for comparison purposes.

#### Dual-channel LSTM network with transfer learning

LSTM is a special type of Recurrent Neural Networks to overcome the vanishing gradient problem when training using gradient descent with backpropagation through time. LSTM can efficiently learn the temporal dependencies and has been successfully used in applications involving signals with temporal memory. In this work, LSTM architecture proposed by [37] is used.

LSTM unit consists of input gate (*i*), input modulation gate (*g*), forget gate (*f*), output gate (*o*), and memory cell ($c_t$ at time step *t*). Before applying the operations in these gates, current feature vector ($\vec {fv}^{(r)}_t$) at time *t* in round *r* is linearly transformed using the following equation:

1 $$\begin{aligned} \vec {x}^{(r)}_t=W_{fx} \vec {fv}^{(r)}_t +b_{fx} \end{aligned}$$

where $\vec {x}^{(r)}_t \in \mathbb {R}^{N_H}$, ${N_H}$ is the number of hidden states and $W_{fx}$ and $\vec {b}_{fx}$ are the weight matrix and bias vector, respectively. The operations in these gates are performed on $\vec {x}^{(r)}_t$ using ${N_H}$ hidden states ($h_{t-1} \in \mathbb {R}^{N_H}$) and internal states ($c_{t-1} \in \mathbb {R}^{N_H}$) from the previous time step as defined below:

2 $$\begin{aligned} i_t=& {} \sigma \left( W_{xi} \vec {x}^{(r)}_t + W_{hi} h_{t-1} +b_i\right) \end{aligned}$$

3 $$\begin{aligned} g_t=& {} \phi \left( W_{xg} \vec {x}^{(r)}_t + W_{hg} h_{t-1} +b_g\right) \end{aligned}$$

4 $$\begin{aligned} f_t=& {} \sigma \left( W_{xf} \vec {x}^{(r)}_t + W_{hf} h_{t-1} +b_f\right) \end{aligned}$$

5 $$\begin{aligned} o_t=& {} \sigma \left( W_{xo} \vec {x}^{(r)}_t + W_{ho} h_{t-1} +b_0\right) \end{aligned}$$

6 $$\begin{aligned} c_t=& {} f_t c_{t-1}+i_t g_t \end{aligned}$$

7 $$\begin{aligned} h_t=& {} o_t \phi \left( c_t\right) \end{aligned}$$

where $W_{ab}$ is a weight matrix ($a=\{x,h\}$ and $b=\{i,g,f,o\}$), and $\sigma$ and $\phi$ are the logistic sigmoid and tanh activation functions, respectively. The output ($\hat{y}^{(r)}$) in many-to-one LSTM network is calculated based on $h_{t}$ of the last LSTM layer and last $\vec {x}^{(r)}$ in round *r* using the following linear transformation:

8 $$\begin{aligned} \hat{y}^{(r)}=W_{hy} h_{t} +b_y \end{aligned}$$

After segmentation and feature extraction (refer to segmentation and feature extraction sections), there were only one long-term feature vector for each 1-min window while there are 12 short-term feature vectors. Therefore, we developed a dual-channel LSTM network to combine the two sets of feature vectors as a strategy to appropriately handle the differences in the number of the short-term feature vectors ($S^{(r)}=[\vec {fv}_1\vec {fv}_2...\vec {fv}_{N_{Ws}^{(r)}}]$) and long-term feature vectors ($L^{(r)}=[\vec {fv}_1\vec {fv}_2...\vec {fv}_{N_{Wl}^{(r)}}]$). This method was based on building a separate LSTM channel on the short-term and long-term sets ($\mathcal {D}_{S}$ and $\mathcal {D}_{L}$, respectively) and then integrating the outcome of the two channels into one UPDRS-III score estimation using a fully connected layer. The feature vectors in both sets were linearly transformed using a fully connected layer to have a depth of $N_{H}$ hidden states in both channels (Eq. 1). The transformed feature vectors $\vec {x}^{(r)}$ were then passed to a many-to-one LSTM network in both channels as shown in Fig. 5a. The hidden states $h_{t}$ from the last feature vector in both channels were then concatenated to create a fusion feature that was passed through a fully connected layer to estimate UPDRS III (Eq. 8).

*Transfer learning:* Due to the limited number of data rounds in the PD dataset used to train the LSTM network, we applied transfer learning to improve the LSTM performance. The LSTM network’s weights to estimate UPDRS III were not randomly initialized; instead, they were transferred from an LSTM network trained to perform activity classification. Next, only the last layer of the LSTM network and the fully connected layers were fine-tuned for estimating UPDRS III. PAMAP2 dataset was used to train the LSTM network for activity classification initially. Note that transfer learning could only be used in the case of the hand-crafted features. Although the sensors in PD and PAMAP2 were placed on the same extremity, the axes’ orientations and the placement on the same extremity were different. Therefore, the learned deep model’s weights on PAMAP2 were not transferable to the PD dataset when the raw signals were used. However, extracting features and averaging them across axes eliminated the effect of having different sensors’ orientation in the PAMAP2 dataset and PD dataset.

#### 1D CNN-LSTM network

We used CNN as a data-driven feature extraction method to explore raw signals. We fed the feature maps of CNN into an LSTM network to model the feature maps’ temporal dependencies and estimate UPDRS III. Our proposed 1D CNN-LSTM is shown in Fig. 5b. It consisted of three convolutional blocks. The first block consisted of two convolutional layers with 32 filters with a width of 8, followed by a max-pooling layer. The second block had the same structure but deeper with 64 filters. The third block had one convolutional filter and a global average pooling layer representing the bottleneck to extract short-term, data-driven features. These features were feed to a many-to-one LSTM network followed by two fully connected layers (96 nodes and one output node) to estimate UPDRS III. Increasing the number of convolutional layers was done by repeating Conv Block-2 multiple times.

Training a good-performing CNN-LSTM model on a relatively limited number of training rounds could be challenging. We applied data augmentation by allowing for a random start for each round of ADL and a 0.5-dropout layer to overcome this challenge. Besides, we proposed a novel two-stage training. In the first stage, a CNN network with a fully connected layer was trained on 5-s windows to estimate UPDRS III while extracting short-term features. The best CNN’s weights selected based on validation data were saved. In the second stage, the fully connected layer of the pre-trained CNN was discarded since they are not extracting new features. Next, the extracted features using the CNN model (i.e., from the global averaging layer) were fed to the LSTM network to estimate UPDRS III for each ADL round.

#### 2D CNN-LSTM network

Many PD symptoms have spectral features such as tremor that manifest in 4–6 Hz and bradykinesia in low frequencies. Therefore, the CNN network can learn new temporal and spectral features if trained on the time–frequency representations of the raw signals. For this purpose, we generated spectrograms by applying a short-time Fourier transform on the 1-min windows and then taking the magnitude. We used a 5-s Kaiser window with 90% overlaps. The spectrograms of the windows from each axes were stacked to construct a time $\times$ frequency $\times$ axes tensor and were fed to a 2D CNN-LSTM network as shown in Fig. 5c. The 2D CNN-LSTM consisted of three convolutional blocks. The first block was two convolutional layers with 32 filters of width five by five, followed by a max-pooling layer. The rest of the architecture of the 2D CNN-LSTM was similar to 1D CNN-LSTM described before except for using filters of size 5 $\times$ 5. In addition, the same two-stage training strategy described before was used to address the limiting training data.

#### The Ensemble Model

We explored the accuracy of UPDRS III estimation by considering the ensemble of the three models we developed. As shown in Fig. 5d, the ensemble of the previous models was performed by averaging the UPDRS-III scores from each model to get one estimation for each round of ADL.

#### Gradient Tree Boosting

Gradient Tree Boosting is a traditional machine-learning method used in practice for solving regression problems [38]. It is based on ensemble of $N_t$ weak regression trees ($\{f_i\}_{i=1}^{N_t}$) to estimate the output $\hat{y}$ or the UPDRS-III score as follows:

9 $$\begin{aligned} \hat{y}\left( \vec {fv}_t\right) =\sum _{i=1}^{N_t} {f_i\left( \vec {fv}_t\right) } \end{aligned}$$

where $f_i(\vec {fv}_t)=w_{q(\vec {fv}_t)}$ is the space of regression tree *i* with *L* leaves, $q(\vec {fv}_t)$ is the structure of the tree that maps $\vec {fv}_t$ to an index represents the corresponding tree leaf, and $w \in \mathbb {R}^L$ is the leaf weights. Learning the regression trees is performed using additive training strategy by learning one tree at each iteration that optimize the objective function which includes the first and second gradient statistics on the loss function.

The short- and long-term feature vectors (refer to the feature extraction section) were combined into one feature vector and were fed into the Gradient Tree Boosting model. For every 5-s segment in a 1-min interval, the long-term feature vectors $\vec {fv}$ were repeated and concatenated with the corresponding short-term feature vectors $\vec {fv}$ to form a matrix of $N_{Ws}$ feature vectors with ($N_{SF}+N_{LF}$) number of features ($SL^{(r)} \in \mathbb {R}^{N_{Ws}^{(r)} \times (N_{SF}+N_{LF})}$). The combined set $\mathcal {D}_{TB}=\{ (SL^{(r)},y^{(r)}) \}_r^{N_R}$ was used to train and test the model. To estimate the average $\hat{y}^{(r)}$ (i. e. UPDRS III) of round *r* during testing, the model first estimate $\hat{y}$ for each of the feature vectors in $SL^{(r)}$, and then they were averaged to get the average $\hat{y}^{(r)}$ (i. e. UPDRS III) for that round.

### Implementation

The UPDRS-III estimation methods were evaluated and compared using the data of 24 PD subjects described in the dataset section using LOOCV. In addition, an inner split was applied on the training data to select a random 20% for validation. The mean and standard deviation of the training data in each cross-validation iteration were calculated and used to normalize the entire data. The developed dual-channel LSTM and CNN-LSTM networks were implemented in TensorFlow [39]. In each cross-validation iteration, the networks were trained for 200 epochs using Adam optimizer [40]. During the training, the depth of the CNN and LSTM networks and filter sizes were optimized by selecting the best performing model on the validation data (i.e. maximum validation $\rho$) then evaluating them on the held-out test data. The depth of the CNNs was increased by repeating Conv Block-2 up to four times. The LSTM hyper-parameters space (number of layers: 1–3 and number of hidden states: 16–224) were searched. Mini-batches of size 2 and learning rate of 1e-3 were used during the training. In each mini-batch, the signals of all the rounds were repeated to have a length equal to the longest round. In addition, before feeding the hand-crafted or data-driven features of each round to the network in each epoch, a random start point was initialized and data prior to the start point was excluded. This augmentation approach was applied to prevent the LSTM network from memorizing the training sequence.

The Gradient Tree Boosting algorithm was implemented using XGboost library [38]. The learning rate was 0.1. A grid search was applied to find the optimal number of regression trees in the range of 10–200 with a step of 20. The tree depth was in the range of 3–10 with a step of 2. The percentage of used-features per tree was in the range of 10–50% with a step of 10%.

## **Supplementary Information**

**Additional file 1.** contains the ensemble model estimations of UPDRS III overtime for all 24 PwPs, and the total UPDRS-III scores before and onehour after taking the PD medications as estimated by the developed single models.

## Data Availability

The PAMAP2 activity dataset is publicly available, and the PD dataset used and analyzed during the current work are available from the corresponding author on reasonable request.

## Abbreviations

PD: Parkinson’s disease
PwP: People with Parkinson’s disease
UPDRS III: Unified Parkinson Disease Rating Scale-part III
LSTM: Long Short-Term Memory
CNN: Convolutional Neural Network
ANN: Artificial Neural Network
SVR: Support Vector Regression
ADL: Activities of daily living
LOOCV: Subject-based, leave-one-out cross-validation
PAMAP2: Public dataset of motion signals
