# Supplementary material for: Ensemble deep model for continuous estimation of Unified Parkinson’s Disease Rating Scale III
Source: Biomed Eng Online. 2021 Mar 31;20:32. doi: 10.1186/s12938-021-00872-w (PMC8010504; doi:10.1186/s12938-021-00872-w)

**Supplementary Online Content for “Ensemble Deep Model for Continuous Estimation of Unified Parkinson’s Disease Rating Scale III”**

**The ensemble model estimations of UPDRS III overtime for all 24 PwPs**

We developed an ensemble of three deep learning models to detect UPDRS-III-related patterns from a combination of hand-crafted features, raw temporal signals, and their time-frequency representation. Supplemental Figure 1 and 2 shows the ensemble model estimations of UPDRS III overtime vs. the gold-standard UPDRS-III scores for all the 24 PwPs.

**The total UPDRS-III scores before and one hour after taking the PD medications**

As shown in Supplemental Figure 3A, a reduction in the gold-standard UPDRS-III score is expected from before to one hour after the medication intake. We investigated whether the estimated scores by the developed single models show similar behavior in the UPDRS III scores as the medication kicks in. The models were Gradient Tree Boosting, dual-channel LSTM with hand-crafted features, dual-channel LSTM with hand-crafted features and transfer learning, 1D CNN-LSTM for raw signals, and 2D CNN-LSTM for time-frequency data. Both the gold-standard and estimated UPDRS-III scores indicate a significant difference after patients take their PD medications as confirmed by a paired t-test with *p*<0.01.

**Supplemental Figure 1:** The ensemble model estimations of UPDRS III overtime vs. the gold-standard UPDRS III for PwPs 1 to 12.


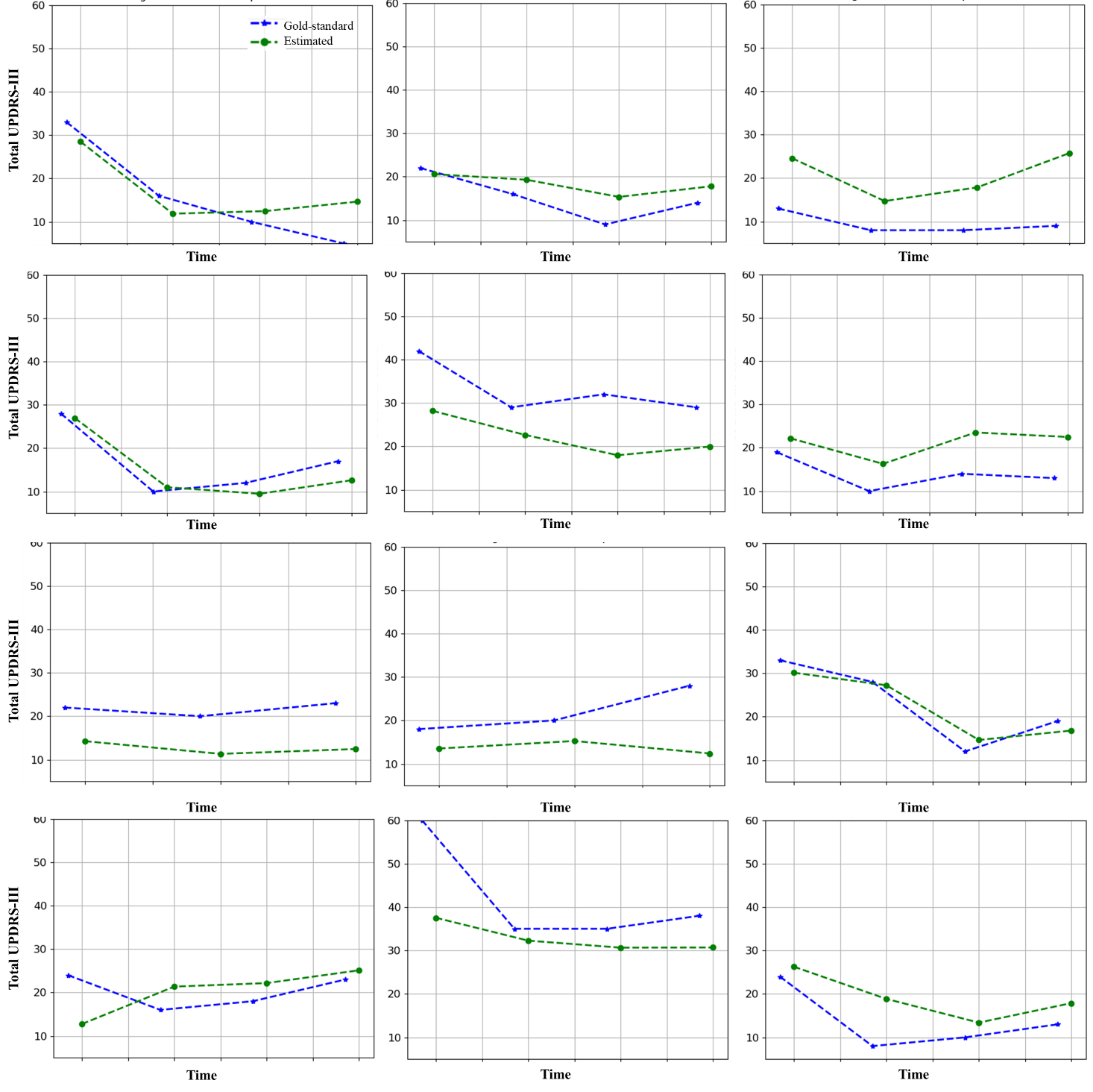


**Supplemental Figure 2:** The ensemble model estimations of UPDRS III overtime vs. the gold-standard UPDRS III for PwPs 13 to 24.


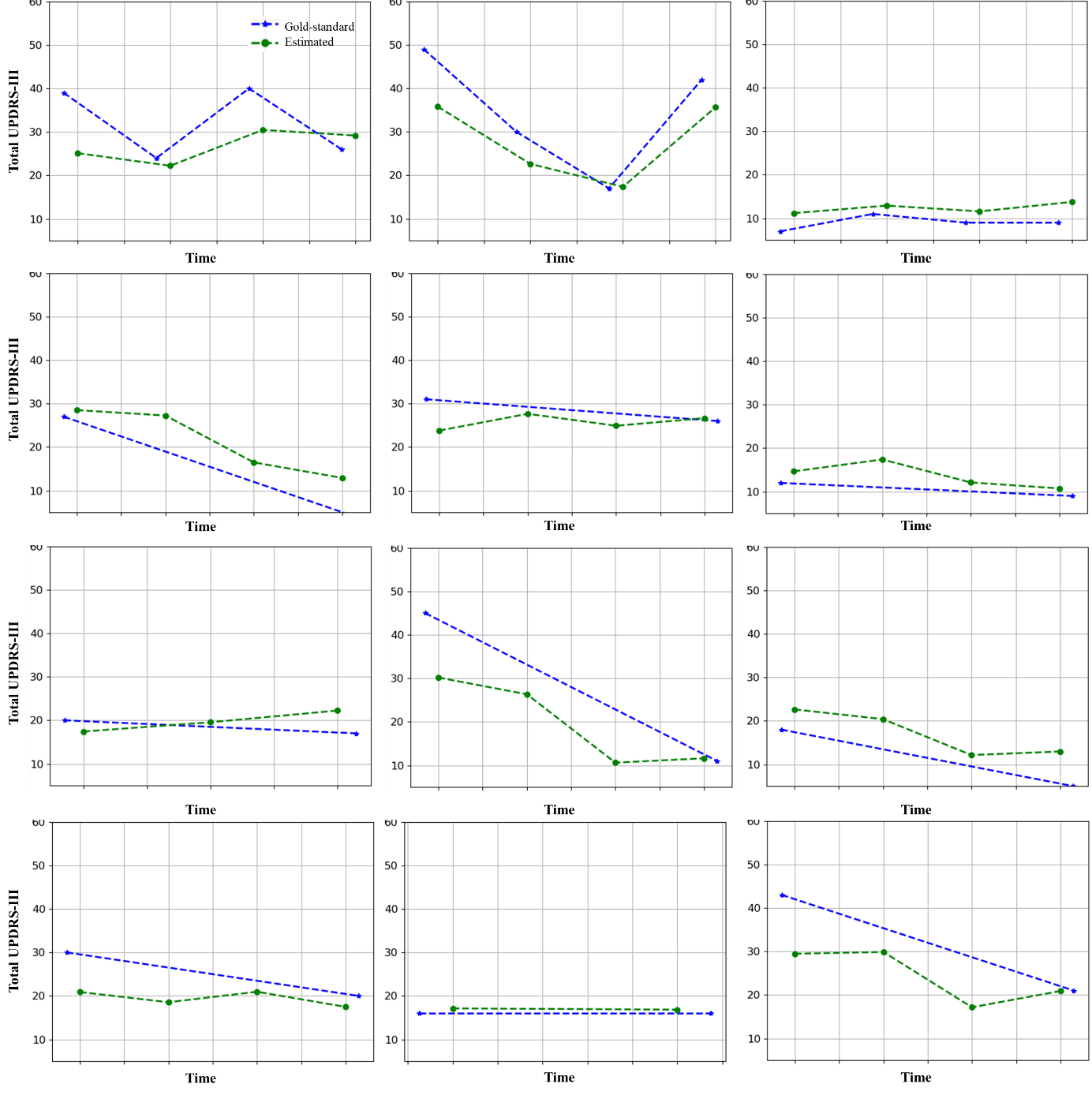


**Supplemental Figure 3:** The total UPDRS-III scores before and one hour after taking the PD medications from gold-standard measurements (A) and the estimations using Gradient Tree Boosting (B), dual-channel LSTM, hand-crafted features (C), dual-channel LSTM, hand-crafted features with transfer learning (D), 1D CNN-LSTM for raw signals (E) and 2D CNN-LSTM for time-frequency data (F). Both the gold-standard and estimated UPDRS-III scores show a significant drop after the PD medication intake (*p*<0.01).


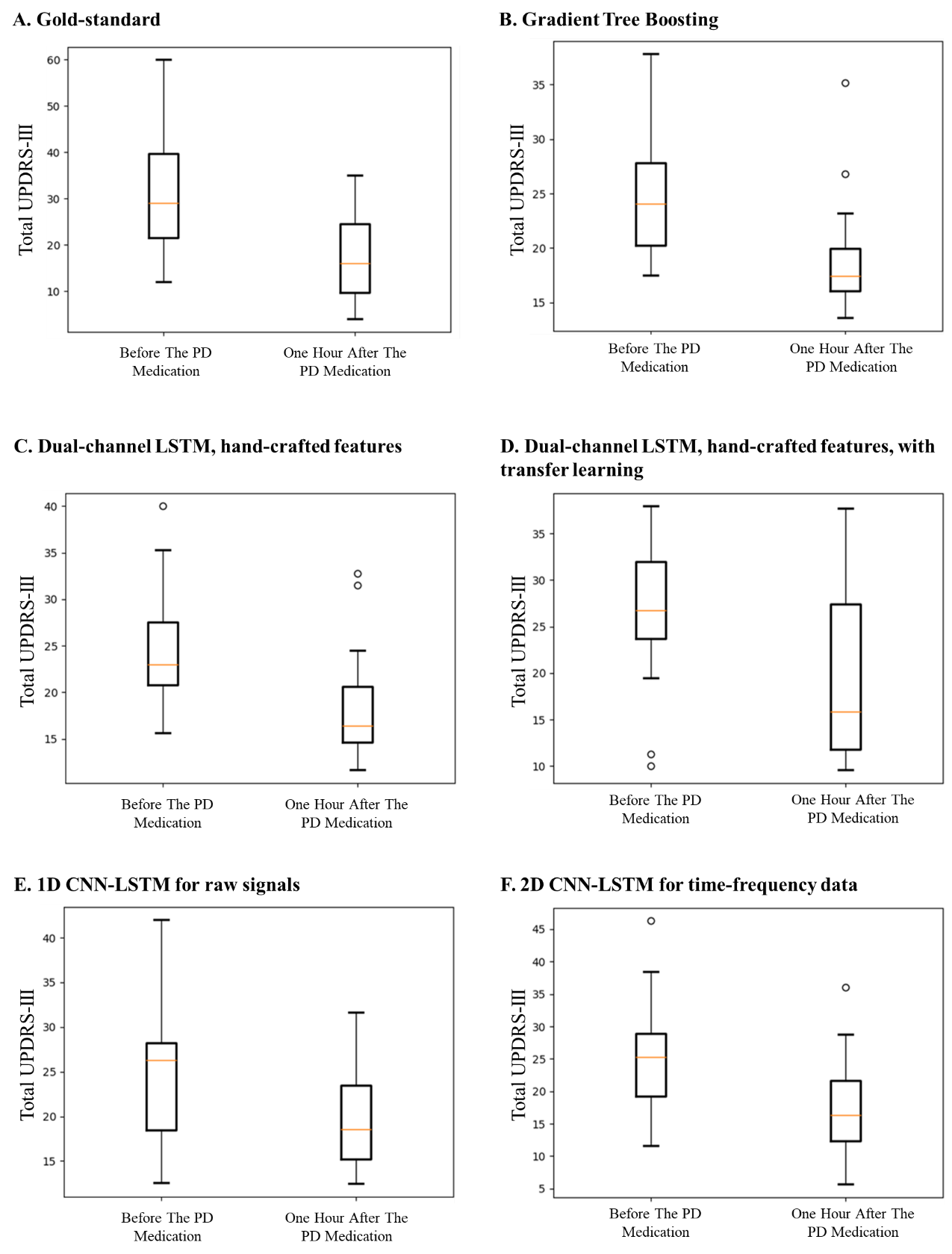

Supplement: Supplementary file 1 — Additional file 1. contains the ensemble model estimations of UPDRS III overtime for all 24 PwPs, and the total UPDRS-III scores before and onehour after taking the PD medications as estimated by the developed single models. [file 12938_2021_872_MOESM1_ESM.docx]
